# Metformin inhibits growth and prolactin secretion of pituitary prolactinoma cells and xenografts

**DOI:** 10.1111/jcmm.13963

**Published:** 2018-10-18

**Authors:** Jun Gao, Yang Liu, Gaijing Han, Kan Deng, Xiaohai Liu, Xinjie Bao, Ming Feng, Yong Yao, Wei Lian, Bing Xing, Xiang Lv, Renzhi Wang

**Affiliations:** ^1^ Department of Neurosurgery Peking Union Medical College Hospital Chinese Academy of Medicine Sciences & Peking Union Medical College Beijing China; ^2^ Head and Neck Surgery Department National Cancer Center & Cancer hospital Chinese Academy of Medical Sciences & Peking Union Medical College Beijing China; ^3^ State Key Laboratory of Medical Molecular Biology Department of Pathophysiology Institute of Basic Medical Sciences Chinese Academy of Medical Sciences & Peking Union Medical College Beijing China

**Keywords:** AMPK, bromocriptine, drug‐resistant prolactinomas, metformin, oestrogen receptor

## Abstract

Metformin (MET) is a diabetes drug that activates AMP‐activated protein kinase (AMPK), and is suggested to have anticancer efficacy. Here, we investigated the role of AMPK signalling in prolactinoma (PRLoma), with particular respect to MET and bromocriptine (BC) as a PRLoma treatment. We analysed AMPK phosphorylation, dopamine D2 receptor (D2R), and oestrogen receptor (ER) expression in both BC‐sensitive and ‐resistant PRLoma samples; effects of the AMPK agonist MET (alone or with BC) on in vitro proliferation and apoptosis, xenograft growth and prolactin (PRL) secretion of BC‐sensitive and ‐resistant cells, and ER expression in xenografts. **S**ome BC‐resistant PRLomas showed high D2R expression but extremely low AMPK activation. MET significantly inhibited proliferation of cultured PRLoma cells; MET + BC notably restrained their PRL secretion. MET + BC further decreased tumour growth and serum PRL levels in xenografts than BC treatment alone. ER was down‐regulated after AMPK activation in both cultured cells and xenografts. Together, we propose that the AMPK signalling pathway down‐regulates ERα and ERβ, and suppresses PRLoma growth as well as PRL secretion. Combined MET + BC is a potential treatment for PRLomas.

## 1 INTRODUCTION

Pituitary adenoma comprises 10%‐15% of all diagnosed intracranial tumours.^1^ PRLoma is the most common type of functional pituitary adenoma, with a prevalence of 100 per 1 million people.^2^ Currently, dopamine agonists such as BC are the primary therapy for PRLoma, with a high probability of controlling tumour size and reducing PRL level. Reportedly, BC normalizes PRL levels in 80%‐90% of cases, and reduces tumour size in ~70% cases.^3^ As in previous studies, we herein define BC resistance as the failure to normalize PRL levels or to reduce tumour size by ≥50%, after taking ≥15 mg/day of BC for at least 3 months.^4^, ^5^ The mechanisms of BC resistance are unclear, although reduced expression of D2R is believed to be the main factor.^3^

The AMPK pathway reportedly mediates proliferation or apoptosis in multiple cancer cell types, including non‐small cell lung cancer,^6^ glioblastoma,^7^ breast cancer,^8^ which implies a complex role for AMPK in tumour cell survival. Depending on cancer type, AMPK functions either as an oncoprotein or a tumour suppressor.^9^ However, the role of AMPK in PRLoma has not been clear.

Activation of AMPK reportedly inhibits mammalian target of rapamycin (mTOR), and suppression of the mTOR pathway would induce autophagy‐dependent cell death in PRLomas.^10^ Notably, AMPK can be activated by AMP‐mimetic 5‐aminoimidazole‐4‐ carboxamide ribonucleoside (AICAR),^11^ and by MET.^8^ The latter is a widely used treatment for type 2 diabetes mellitus.^12^ The function of MET is primarily associated with its activity on cellular energy metabolism. By restraining Complex I in the mitochondrial respiratory chain, MET generates cellular energy stress and thus activates AMPK.^13^ Accumulating evidence supports an anticancer effect of this drug.^14^

To determine whether the AMPK pathway affects PRLoma development, we measured AMPK phosphorylation in human primary PRLoma samples, and investigated the effect and downstream effectors of AMPK agonist MET in PRLoma using BC‐sensitive MMQ cells and BC‐resistance GH3 cells and their xenografts as models. Our working hypotheses are that the AMPK pathway is a potential therapeutic target for PRLoma, and MET combined with BC is a potential treatment for PRLoma.

## 2 RESULTS

### 2.1 Bromocriptine resistance was associated with down‐regulated AMPK activity and high oestrogen receptor expression

**Figure 1:**
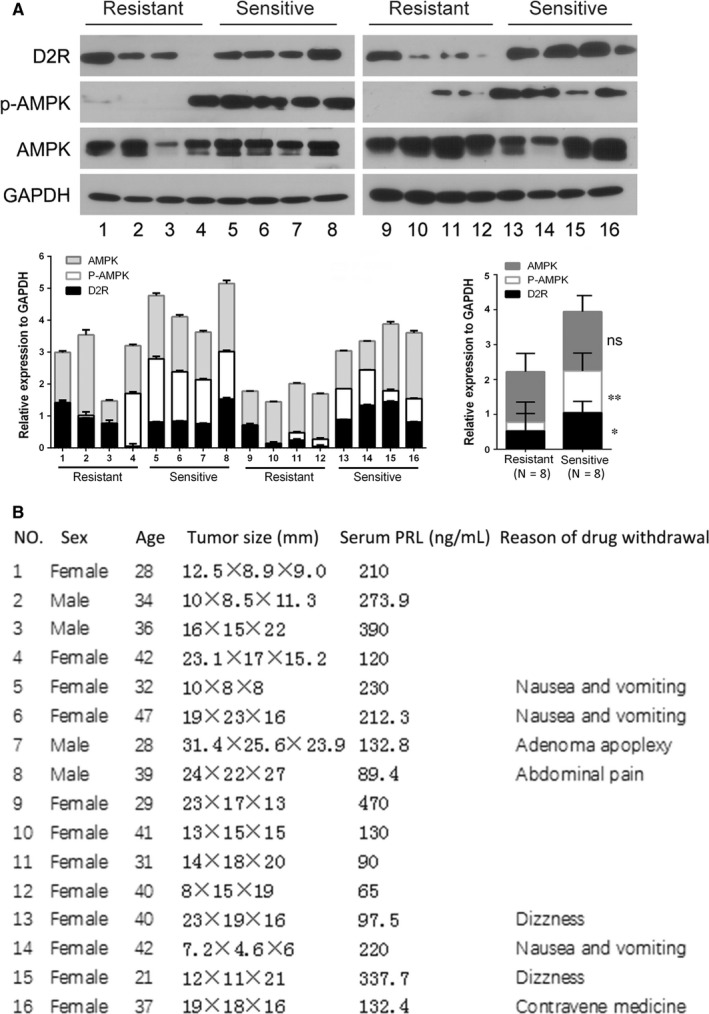
AMPK phosphorylation is significantly decreased in dopamine D2 receptor‐positive bromocriptine‐resistant prolactinoma. A, Western blotting assay of dopamine D2 receptor (D2R), AMPK and phosph‐AMPK (p‐AMPK) levels in prolactinomas from bromocriptine‐resistant (NO.1‐4, 9‐12) and ‐sensitive (NO.5‐8, 13‐16) patients. The lower left panel shows quantification of the western blotting assay and the lower right panel shows statistic analysis of the quantification (Student's *t* test, ns, non‐significant, *P <* *0.05, **P <* *0.01). B, General information of the patients who contributed to the prolactinoma specimens analysed in (A)

To explore whether AMPK is related to drug‐sensitivity in PRLomas, we collected samples of BC‐sensitive and ‐resistant PRLomas and examined them for expression of D2R, and AMPK protein and phosphorylation levels. As expected, D2R expression was generally higher in the BC‐sensitive group (Figure 1A), although in the BC‐resistant group, Patients 1, 2, 3, and 9 also showed high D2R levels. AMPK activity, as represented by phosphorylated AMPK (p‐AMPK), was significantly higher in the BC‐sensitive group than in the BC‐resistant group. Notably, the four BC‐resistant patients with high D2R levels (Patients 1, 2, 3, and 9) all had almost undetectable p‐AMPK levels (Figure 1A). General clinical data of the 16 PRLoma patients were listed in Figure 1B.

**Figure 2:**
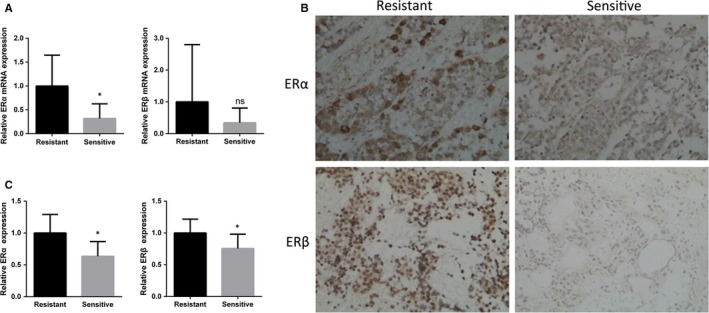
RT‐PCR A, and immunostaining. B, C, analysis of ERα and ERβ expression in prolactinomas from bromocriptine‐sensitive and ‐resistant patients. (Student's *t* test, ns, non‐significant, *P <* *0.05)

As ER expression has been shown to contribute to drug resistance in PRLoma,^15^, ^16^ we measured ER levels in the human PRLoma samples. RT‐PCR analysis showed that ERα expression was significantly higher in drug‐resistant samples than in the drug‐sensitive ones. Median expression of ERβ tended to be higher in the resistant group, although not significantly so (Figure 2A). Immunohistochemistry confirmed higher ERα and ERβ levels in drug‐resistant patients (Figure 2B,C).

### 2.2 MET activates AMPK in PRLoma cells and inhibits their growth and prolactin secretion

**Figure 3:**
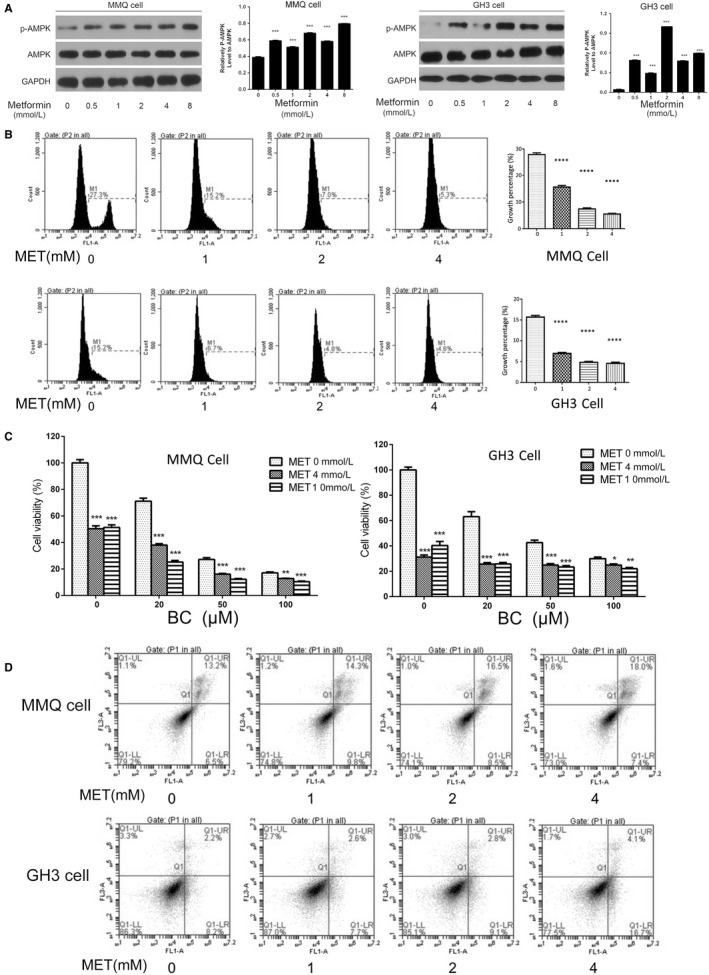
Metformin activates AMPK, suppressed proliferation, but has no significant effect on apoptosis of both the bromocriptine‐sensitive MMQ cells and the bromocriptine‐resistant GH3 cells. A, MMQ and GH3 cells were treated with metformin of indicated concentrations for 24 hours before subjected to western blotting analysis of AMPK and pAMPK levels. Quantification of the western blotting assay is shown in graphs to the right of each image. (Student's *t* test, ***P < 0.001 vs blank) B, EdU cell cycle analysis showed markedly repressed proliferation of MMQ and GH3 cells upon the treatment of metformin at indicated concentration. (One‐way Anova analysis, ****P <* *0.0001) C, CCK‐8 assay of GH3 and MMQ cells treated with different combination of MET and BC. (Student's *t* test, *P <* *0.05, **P <* *0.01,***P <* *0.001 vs “MET 0 mM” group) D, Annexin V‐FITC analysis detected no significant changes in apoptosis of MMQ and GH3 cells after treated with Metformin of different dosages

We used MET to activate AMPK in BC‐sensitive (D2R‐positive) MMQ cells and BC‐resistant (D2R‐negative) GH3 cells. Obviously, increased AMPK phosphorylation was detected in both cell lines (Figure 3A). EdU cell cycle analysis revealed that MET markedly suppressed proliferation of both cell lines (Figure 3B). CCK‐8 assay confirmed crippled cell number of GH3 and MMQ upon MET treatment, and showed an additive effect between MET and BC selectively in the D2R‐positive MMQ cell (Figure 3C). AMPK activation also slightly increased proportions of Annexin V‐positive cells in both cell lines, but the difference is not significant (Figure 3D).

**Figure 4:**
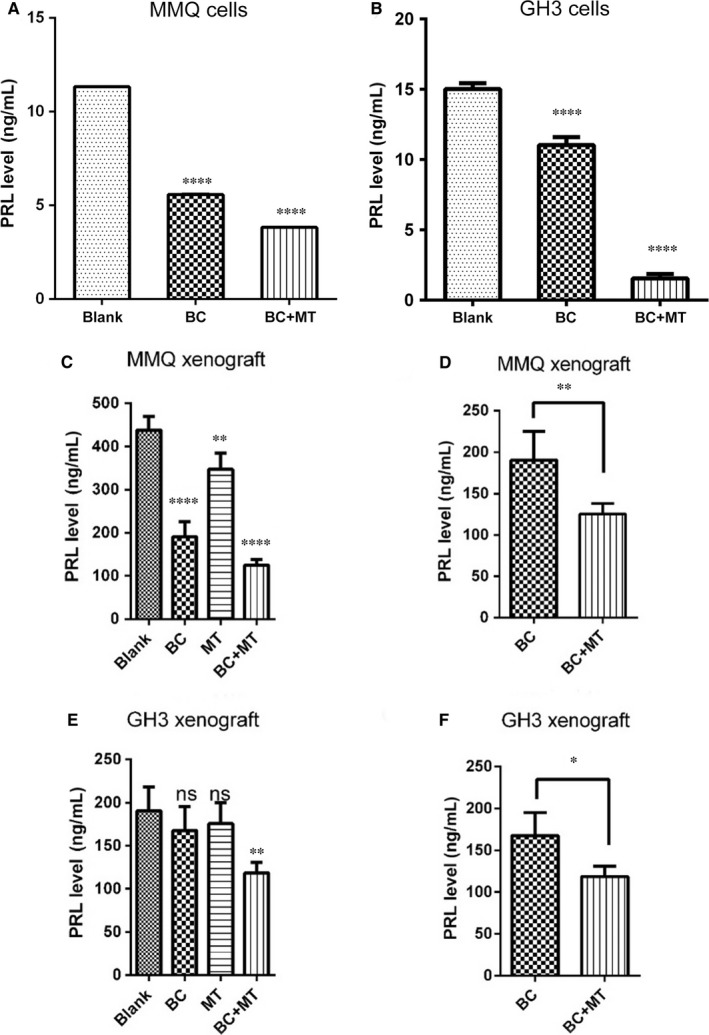
Combined usage of Metformin with BC further reduced the PRL secretion of MMQ and GH3 cells, and their xenografts. A, B, PRL Levels in cell culture supernatants of MMQ (A) and GH3 (B) was determined by ELISA assays before and after the treatment of indicated drugs. BC: bromocriptine (0.4 mmol/L), MT: Metformin (4 mmol/L). (One‐way ANOVA analysis, ****P <* *0.0001) C, F, Plasma PRL levels of MMQ (C) and GH3 (E) xenograft mice in different drug group were examined by ELISA assays (One‐way ANOVA analysis, ns, non‐significant, **P < 0.01, ****P < 0.0001). Combined effects of MET and BC vs BC treatment alone in MMQ (D) and GH3 (F) xenograft mice were highlighted (post hoc pairwise comparison test, ns: non‐significant, *P <* *0.05, **P <* *0.01 vs blank if not specifically indicated)

Prolactin (PRL) levels in cell supernatants were then measured before and after treatment with different combinations of BC and MET. We found BC used alone reduced PRL in both MMQ and GH3 cells (Figure 4A,B); as expected, MMQ cells were more sensitive to BC. Importantly, PRL level is greatly decreased after combined BC + MET treatment, indicating that AMPK activation enhances the effect of BC in cellular PRLoma models.

### 2.3 MET inhibits growth and prolactin secretion in PRLoma xenografts

**Figure 5:**
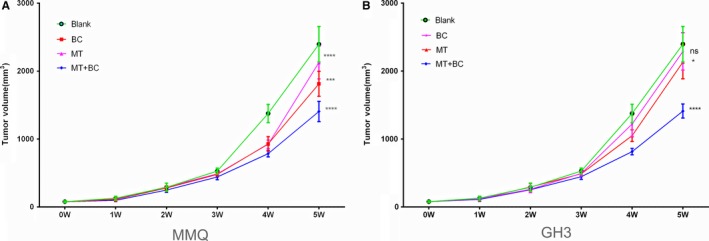
Combined usage of MET with BC further reduced the xenografts growth of MMQ cells and GH3 cells. MMQ A, and GH3 B, xenografted mice were daily treated with saline (blank), 400 mg/kg bromocriptine (BC), 500 mg/kg metformin (MT), or 500 mg/kg metformin in combination with 400 mg/kg bromocriptine (MT + BC), respectively, for five successive weeks. Tumour volume in different drug groups (N = 5 for each group) was measured weekly during the 5‐week period of observation. (Two‐way ANOVA analysis, ns, non‐significant, *P <* *0.05, ***P <* *0.001, ****P <* *0.0001 vs blank if not specifically indicated)

We next assessed the effect of MET on PRLoma growth in a xenograft mouse model. Both BC and MET treatments repressed growth of MMQ xenograft tumours, and slightly shrank GH3 tumours; combined BC + MET further shrank both MMQ and GH3 xenograft tumours to more than 40% (Figure 5). Similarly, plasma PRL levels in the xenografted mice were reduced by MET or BC treatment alone, especially with MMQ xenografts (Figure 4C,E). The combined MET + BC treatment resulted in notably lower plasma PRL levels in both MMQ and GH3 xenograft mice (Figure 4C,E; highlighted in Figure 4D,F). These results showed that MET usage improved the effect of BC on PRLoma in mouse model.

### 2.4 MET reduces ER expression in PRLoma cells and xenografts

**Figure 6:**
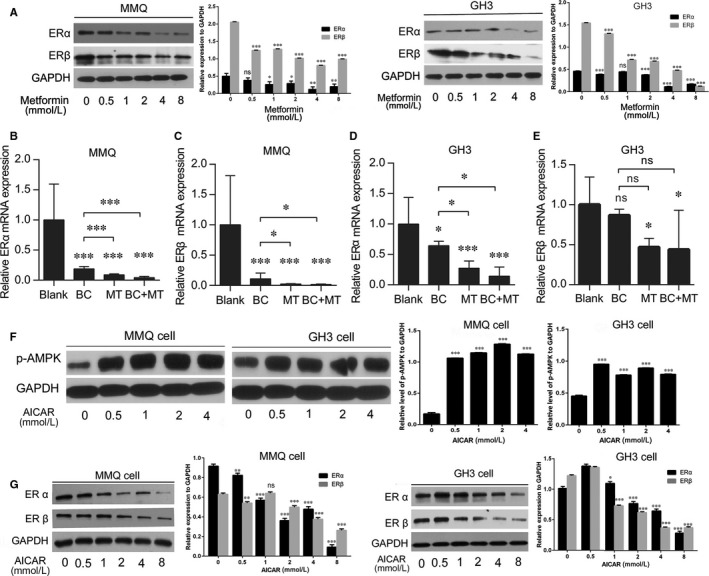
Both metformin and AICAR reduced ER expression in MMQ and GH3 cells. A, ERα and ERβ expression in in vitro cultured MMQ and GH3 cells were detected by western blotting assays before and after the treatment of metformin at indicated concentrations. Quantification of the western blotting assay is shown in graphs to the right of each image. (Student's *t* test, ***P <* *0.001 vs blank) B‐E, MMQ and GH3 xenograft mice were daily treated with saline (blank), 400 mg/kg bromocriptine (BC), 500 mg/kg metformin (MT) or 500 mg/kg metformin in combination with 400 mg/kg bromocriptine (MT + BC), respectively, for five successive weeks. ERα and ERβ mRNA expression in different drug groups of MMQ (B:ERα, C:ERβ) and GH3 (D:ERα, E:ERβ) xenograft tumours were detected by RT‐PCR (N = 5 for each group). Metformin inhibits ERα and β expression in both MMQ and GH3 xenografts with or without combined treatment of bromocriptine. (One‐way ANOVA analysis, ns, non‐significant, *P <* *0.05, ***P <* *0.001 vs blank if not specifically indicated) F, G, western blotting assays detected F, increased AMPK phosphorylation and G, decreased ERα and ERβ expression in AICAR treated GH3 and MMQ cells. Quantification of the western blotting assay is shown in graphs to the right of the images (Student's *t* test, ns, non‐significant, *P <* *0.05, **P <* *0.01, ***P <* *0.001 vs blank)

ER is associated with GH3 cell proliferation,^17^ and is expressed at higher levels in the BC‐resistant human specimens. In the present study, we investigated whether MET affects ER expression in PRLoma cells. Western blotting assays showed that MET reduced ERα and ERβ expression in both BC‐sensitive MMQ cells and BC‐resistant GH3 cells in a dose‐dependent manner. (Figure 6A). Similarly, semi‐quantitative real‐time PCR showed MET to markedly decrease ERα and ERβ mRNA levels in both MMQ and GH3 xenografts (Figure 6B,C,D,E).

To determine whether AMPK activation is important for MET‐induced ERα and ERβ down‐regulation, another AMPK agonist AICAR was applied to GH3 and MMQ cells. pAMPK level in the cells was increased as expected, accompanied by a notable decrease of ERα and ERβ expression, supporting that AMPK signalling participates in the suppression of ER expression (Figure 6F,G).

**Figure 7:**
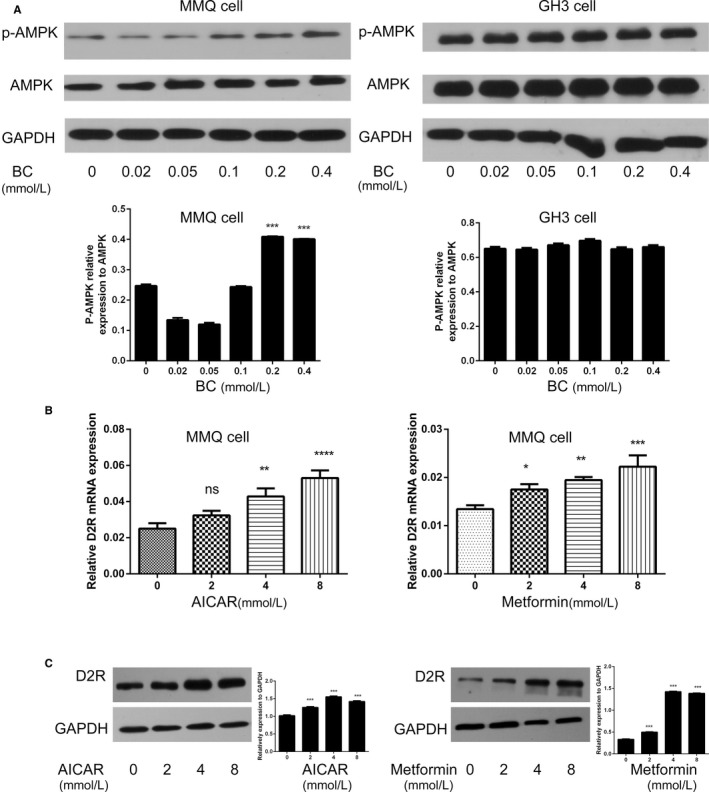
The mutual stimulative effects of bromocriptine/D2R and AMPK signalling pathways. A, Bromocriptine stimulated AMPK phosphorylation in D2R‐positive MMQ cell, but not in the D2R‐negative GH3 cell. Quantification of the western blotting assay is shown in graphs below each image. (Student's *t* test, ***P <* *0.001 vs blank) B, C, Both AICAR and Metformin treatment led to D2R up‐regulation in MMQ cells at B mRNA and C protein levels. Quantification of the western blotting assay is shown in graphs to the right of each image. (B: One‐way ANOVA analysis, C: Student's *t* test, ns, non‐significant, *P <* *0.05, **P <* *0.01,***P <* *0.001, ****P <* *0.0001 vs blank if not specifically indicated)

BC treatment also reduced ERα and ERβ expression in PRLoma xenografts, especially in the sensitive MMQ tumours. Interestingly, we found that BC increases pAMPK level in MMQ but not in GH3 cells (Figure 7A), which may partially underlie the sensitive response of ER expression to BC in MMQ cells. Meanwhile, AMPK‐independent effects of BC and/or D2R activation on ER expression (especially ERα) still pending further investigation. Conversely, both MET and AICAR treatments were found promote D2R expression in the D2R‐positive MMQ cells (Figure 7B,C). Notably, combined BC + MET down‐regulated *ER* mRNA levels were more than BC treatment alone, although further decrease of *ER*β expression was not significant in GH3 tumours.

Altogether, these results suggest that MET targets PRLomas by stimulating AMPK signalling and reducing ER expression. The suppressed AMPK signalling may be related to the BC‐resistance of some PRLoma patients. Moreover, the mutual promotion between D2R and AMPK signalling in MMQ cells may contribute to a better combined effect of MET and BC in restraining PRLomas.

## 3 DISCUSSION

In the present study, we found that AMPK signalling was suppressed in the D2R‐positive BC‐resistant human PRLomas. The AMPK activator MET inhibited proliferation of both BC‐sensitive (MMQ) and ‐resistant (GH3) PRLoma cells. Moreover, MET + BC treatment showed improved effect, compared to BC treatment alone, in arresting growth of xenografts of both cell lines, and reducing their PRL secretion, both in vitro and in the xenografted mice. Mechanistic studies suggest that MET and AMPK activation down‐regulates ER, which may contribute to its anti‐PRLoma activity.

Treatment with dopamine agonists is currently the first choice for PRLomas because they can normalize PRL levels, reduce the volume of tumour, and restore gonadal function.^18^, ^19^ Reportedly, however, about 25% of patients are resistant to BC, and 10% are resistant to cabergoline.^20^ While the response of PRLomas is shown closely related to D2R expression level,^21^ cases existed that PRLomas of high D2R level are resistant to dopamine agonist,^22^ supporting the involvement of other factors in BC resistance.^23^ To better characterise BC‐resistant PRLomas, we followed strict criteria in patient selection in the present study. All patients had taken BC regularly for at least 3 months and been closely followed up. Eight BC‐sensitive patients who had severe drug side effects or strongly refused further medication contributed to the precious surgical specimens of drug‐sensitive PRLoma. The BC‐resistant tumours were from eight age‐ and sex‐matched patients, among them four were shown to express high level of D2R. Interestingly, we found that AMPK was activated in all our BC‐sensitive PRLoma specimens, but severely suppressed in the D2R‐positive BC‐resistant ones. The data from primary PRLoma specimens therefore suggest a role of AMPK activity in regulating PRLoma growth and its BC‐resistance.

Two rat PRLoma cell lines, the BC‐sensitive MMQ and the BC‐resistant GH3, were used in this study to evaluate the role of AMPK signalling and the efficacy of MET in restraining PRLoma. GH3 cells are known as somatolactotroph cells. Previous studies reported a role of AMPK activator MET and AICAR in suppressing proliferation and growth hormone secretion of GH3 as a model of growth hormone‐secreting pituitary adenoma, but observed different effect of the drug on cell apoptosis.^24^, ^25^, ^26^, ^27^ How MET and AMPK activation may affect PRLoma, eg, the PRL secretion and the phenotype of other PRLoma cells remain largely unclear. We showed that MET inhibits the growth of GH3 and MMQ cells, as well as their xenografts, and provided evidence that MET significantly reduce PRL secretion of the two cells in vitro and in xenograft tumours. Meanwhile, we detected no significant pro‐apoptotic effect of the drug on both cell types, consistent to the report by Faggi L. et al.^26^ Moreover, our results supported advantage of combined MET + BC usage over BC alone for PRLoma treatment, considering cell proliferation, PRL secretion, and xenograft growth. In support of this possibility, we recently witnessed cases of two patients with refractory PRLoma who significantly improved after a regimen of combined MET + BC.^28^

Mechanistically, previous work has noted a sex‐dependent effect of MET on serum PRL levels, and suggested the involvement of thyroid's secretory axis as well as the central dopaminergic transmission.^29^ We found here for the first time that MET suppresses ERα and ERβ expression in PRLoma cells and their xenografts. Studies have suggested critical roles for oestrogen and its receptors in the development of PRLoma.^16^, ^30^, ^31^, ^32^, ^33^, ^34^, ^35^, ^36^, ^37^ Oestrogens retard the effects of dopamine agonists, with direct action on *PRL* gene transcription, stimulation of mitotic activity, and modulation of the inhibiting effect of dopamine on *PRL* gene transcription.^30^ Prolonged oestrogen administration in animals, and also in men, induced the appearance of pituitary tumours, especially PRLomas.^31^, ^32^ Moreover, approximately 30% of patients with PRLomas experience increased tumour volume during pregnancy due to increased oestrogen levels.^33^ Clinical studies have confirmed that ER expression is positively correlated with tumour PRL level^34^ and is significantly increased in invasive PRLomas.^35^ Conversely, ER antagonist fulvestrant showed reduced size and PRL secretion of PRLomas,^16^, ^36^ and tamoxifen was successfully used to treat a dopamine agonist‐resistant PRLoma.^37^ These data, together, suggest that ER may be responsible for the anti‐PRLoma activity of MET and for its reported sexual dimorphism of the anti‐PRLoma activity.^29^

**Figure 8:**
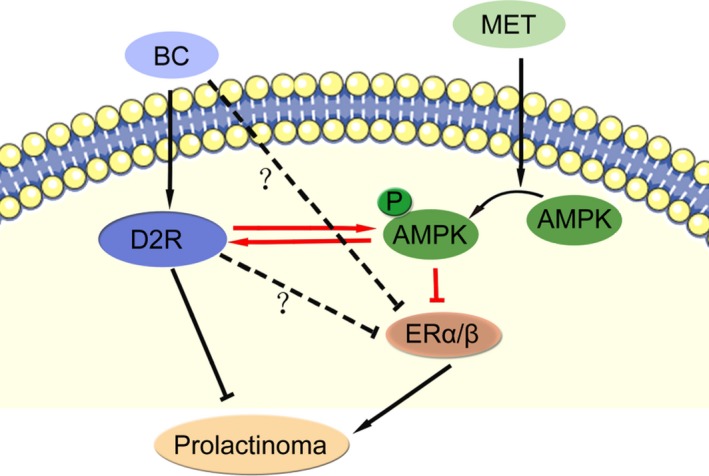
Schematic representation of metformin/AMPK/ER signalling pathway and its potential crosstalk with bromocriptine/D2R pathway in prolactinoma treatment. Red arrow/line refers to regulatory relationship proposed in this study. Dashed line with question mark refers to potential AMPK‐independent pathway for BC/D2R to regulate ER expression

In addition to MET, we showed that AMPK agonist AICAR also decreased ER expression in both GH3 and MMQ cells. Moreover, we found in the D2R‐positive MMQ cells a mutual stimulative relationship between BC/D2R signalling and AMPK activation. BC treatment suppresses ERα and ERβ expression in MMQ and its xenografts, the suppression is much weaker in D2R‐negative GH3 cells. Consistently, oral administration of BC was found to significantly decrease ER levels in patients with sensitive PRLomas.^38^ The observations support that AMPK activation inhibits of ERα and ERβ expression in PRLomas. A schematic representation of the MET/pAMPK/ER signalling pathway and its potential crosstalk with BC/D2R pathway in PRLoma treatment is shown in Figure 8. The physiological relevance and detailed signalling pathway for the interplays of D2R and pAMPK, and for that from pAMPK to ER, are pending further exploration.

Together, our results suggest that the suppressed AMPK signalling pathway contributes to drug resistance of pituitary PRL adenomas and represents an effective target for BC‐resistant PRLoma. We propose that therapeutic effect of BC can be improved by combined usage of the AMPK activator MET in treating pituitary PRLoma—partially by reducing ERα and ERβ expression. Our results imply a strong translational possibility in the treatment of PRLomas.

## 4 MATERIALS AND METHODS

### 4.1 Human PRLoma tissue specimens

We collected samples of PRLoma (n* *=* *16, including eight BC‐sensitive samples and eight BC‐resistant samples) from patients who had undergone resections for PRLomas in the Neurosurgery Department of Peking Union Medical College Hospital, from 2012 to 2014. This research was approved by the Clinical Medicine Ethics Committee of the Peking Union Medical College Hospital.

### 4.2 Immunohistochemistry staining and Image analysis

Paraffin sections of PRLoma tissues were first deparaffinised in xylene and rehydrated. Endogenous peroxidase activity was quenched with 3% (v/v) H_2_O_2_ for 10 minute, followed by wet heat‐induced epitope retrieval in citrate buffer. The slides were incubated with anti‐ERα or anti‐ERβ antibody (Santa Cruz, Texas, USA, #sc‐542, #sc‐8974) overnight at 4°C and then with a HRP‐conjugated secondary antibody at 37°C for 30 minute before subsequent staining with diaminobenzidine and haematoxylin. The results of immunohistochemistry were semi‐quantitatively analysed by Image‐pro plus software (Media Cybernetics). In each case, six high power fields were selected under microscope. The mean optical density was measured according to the tissue area and intensity, and analysed by Student's *t* test.

### 4.3 Semi‐quantitative real‐time polymerase chain reaction

Total RNA was extracted from human PRLoma tissues and xenograft tumours using Trizol Reagent (Invitrogen, Carlsbad, CA, USA), according to the product manual. We reverse‐transcribed 2 μg RNA from each sample by extension of oligo primers (TaKaRa) using M‐MLV reverse transcriptase (New England Biolabs) following the manufacturer's protocol. Real‐time PCR was performed on a Bio‐Rad IQ5 cycler using a SYBR Green reaction mix (Takara). Primers are listed below. Relative expression levels were standardised to *GAPDH* as internal control for all real‐time PCR assays.

Hum‐ERα Forward Primer: TCTGTCTCCTGCATACACTC

Reverse Primer: GGGAATCCTCACGCTTAG

Hum‐ERβ Forward Primer: GCTTTGGTTTGGGTGATTG

Reverse Primer: CCGAGTTGATTAGAGGGTC

Hum‐GAPDH Forward Primer: TGTGGGCATCAATGGATTTGG

Reverse Primer: ACACCATGTATTCCGGGTCAAT

Rat‐ERα Forward Primer:GCTCCTAACTTGCTCTTGG

Reverse Primer:GGACTCGGTGGATGTGGT

Rat‐ERβ Forward Primer:TCTCCTTTAGCGACCCA

Reverse Primer:ACGCCGTAATGATACCC

Rat‐GAPDH Forward Primer:TTCTTGTGCAGTGCCAGCCTCGTC

Reverse Primer:TAGGAACACGGAAGGCCATGCCAG

### 4.4 Cell lines

Rat MMQ cells, GH3 cells, F12 medium, and F10 medium were obtained from the Cell Center, Peking Union Medical College. MMQ cells were cultured in F12 medium supplemented with 2.5% foetal bovine serum (Gibco, USA), 15% horse serum (Gibco), 100 U/mL penicillin (Gibco), and 100 U/mL streptomycin (Gibco) in a humidified incubator at 37°C with 5% CO_2_. GH3 cells were maintained under the same conditions, except that F10 was used as the culture medium.

### 4.5 Pharmacological studies in vitro

MET (No. D150959) was purchased from Sigma‐Aldrich (St. Louis, MO, USA). Bromocriptine mesylate was provided by Gedeon Richter Plc (Budapest, Hungary). We prepared stock solutions of MET (100 mmol/L, dissolved in water) and BC (10 mmol/L, dissolved in dimethylsulfoxide [DMSO]). The drugs were then diluted in culture medium to indicated concentrations upon usage. Treated and control groups were cultured in a humidified incubator at 37°C with 5% CO_2_ for 24 hours.

### 4.6 Cell proliferation assay

Effects of BC, MET, and combined BC + MET on proliferation of MMQ and GH3 cells in vitro were assessed by CCK‐8 (Dojindo Lab, Kumamoto, Japan) or EdU (Life technologies, Gaithersburg, USA) assay as specifically indicated. For CCK‐8 assay, 3 × 10^4^ GH3 or MMQ cells were seeded per well in a 96‐well plate, and incubated with different combination of BC and MET for 48 hour. Ten microlitres of CCK‐8 reagent was then added per well and cell absorptions at 450 nm were measured 2 hours later. EdU assays were performed according to manufacturer's instructions. Briefly, 1 × 10^6^ GH3 or MMQ cells were seeded per well in a 6‐well plate and cultured with different concentrations of MET for 24 hours. Stock EdU solution was then added to the cell medium to a final concentration of 10 μmol/L. The cells were collected 2‐4 hours later, washed with PBS containing 1% BSA at 4°C for three times, and resuspended in 100 μL Click‐iT buffer to fix for 15 minute. Cells were washed again with PBS containing 1% BSA for three times before transferred to 100 μL 1 × Click‐iT saponin‐based permeabilisation and wash reagent for 15 minute. Finally, 0.5 mL of fleshly formulated Click‐iT Plus reaction cocktail were added and the cells were incubated in dark for 30 minute at room temperature. After three additional washing in the 1 × Click‐iT saponin‐based permeabilisation and wash reagent, the cells were subjected to cytometer analysis at 530 nm and the data were analysed with CFlow Plus analysis software.

### 4.7 Flow cytometry analysis of cell apoptosis

Apoptosis assays were performed using the Annexin V‐FITC Apoptosis assay kit (Biosea, China). MMQ and GH3 cells were treated for 24 hours with control solvent or MET (0, 0.5, 1, and 2 mmol/L). The cells were then collected, washed twice with PBS and resuspended in 200 μL binding buffer containing 5 μL of Annexin‐V‐FITC. Cells were stained with 10 μL propidium iodide and incubated in dark at room temperature for 25 minutes, and assayed with a FACS flow cytometer (Accuri Cytometers, Ann Arbor, MI, USA). Data were analysed with CFlow Plus analysis software.

### 4.8 Western blotting analysis

AMPK and ACC antibody sampler kits were purchased from Cell Signalling (Massachusetts, USA). Anti‐GAPDH antibody from Sigma (Shanghai, China) and anti‐D2R, anti‐ERα and anti‐ERβ antibodies were purchased from Santa Cruz (Texas, USA). For cell experiments, 1 × 10^7^ cells were harvested, washed twice with ice‐cold PBS, lysed in RIPA lysis buffer with 100 mmol/L phenylmethanesulfonyl fluoride, and then centrifuged (12 000 rpm; 4°C; 20 minute) to collect the supernatants.

Samples of xenograft tumours and human PRLomas, were homogenised on ice in RIPA lysis buffer containing 100 mmol/L phenylmethanesulfonyl fluoride. Supernatants were collected after centrifugation.

We separated 10 μg of extracted protein on 12% SDS‐PAGE gel, which was transferred onto an Immobilon‐P membrane. Membranes were blocked in Tris‐buffered saline with 5% non‐fat milk, then probed with designated primary antibodies overnight at 4 °C and incubated with the relevant peroxidase‐conjugated secondary antibody (1:5000) for 1 hour at room temperature. Next, membranes were washed and visualised with an enhanced chemiluminescence system. The AMPK, D2R, ERα, and ERβ expression levels were quantified and normalised to the GAPDH control.

### 4.9 PRLoma xenografts

All procedures were performed according to a protocol approved by the Institutional Animal Care and Use Committee of Peking Union Medical College Hospital. Six‐week‐old female BALB/c nude mice were obtained from the Animal Center, Peking Union Medical College Hospital (Beijing, China). We mixed 100 μL MMQ cells or GH3 cells (2 × 10^7^/mL) with 100 μL Matrigel. Mixtures were injected into the right groins of mice. Tumour volume was calculated as Volume (mm^3^) = Length × Width^2^ × 0.5. When tumour volume reached ~50 mm^3^ in size, mice were randomised into four groups: Group A (controls; treated with 200 μL saline); Group B (400 mg BC/kg only); Group C (500 mg MET/kg only); and Group D (400 mg BC/kg + 500 mg MET/kg). Tumour growth in each drug group (N = 5) were recorded weekly. Five weeks later animals werekilled. Blood was drawn from the retro‐orbital sinus, followed by PRL assay. Tumours were harvested, followed by photography and western blotting analysis of ERα and ERβ expression.

### 4.10 Hormone secretion analysis

For MMQ and GH3 cells, supernatants were collected before or after drug treatment dependent on the experiments. For xenografted animals, blood was collected and centrifuged at 3300 rpm for 10 minute at 4°C to separate plasma; PRL levels in supernatant and plasma were determined with an enzyme‐linked immunosorbent assay kit from USCN Life Science Inc. (Wuhan, China).

### 4.11 Statistical analysis

All data are presented as means ± SD. The statistical analyses were performed using one‐way analysis of variance (ANOVA), two‐way ANOVA, Student's *t* test or post hoc pairwise comparison test as indicated. Analyses were conducted on GraphPad Prism 6 (GraphPad Software, Inc., La Jolla, CA, USA).

## CONFLICT OF INTEREST

The authors claim no conflict of interest.

## AUTHOR CONTRIBUTIONS

JG and YL performed most of the experiments. KD and XHL assisted in the mice experiments. GJH, XJB, and MF contributed to the cell‐related experiments. YY, WL and BX helped to the collection of specimens. XL and JG participated in the data interpretation and manuscript preparation. JG, XL, and RZW conceived the study, designed the experiments and revised the manuscript.
